# Role of high mobility group box protein 1 (HMGB1) in peripheral blood from patients with multiple sclerosis

**DOI:** 10.1186/s12974-015-0269-9

**Published:** 2015-03-11

**Authors:** Sunny Malhotra, Nicolas Fissolo, Mar Tintoré, Ana Cristina Wing, Joaquin Castilló, Angela Vidal-Jordana, Xavier Montalban, Manuel Comabella

**Affiliations:** Servei de Neurologia-Neuroimmunologia, Centre d’Esclerosi Múltiple de Catalunya (Cemcat), Institut de Receca Vall d’Hebron (VHIR), Hospital Universitari Vall d’Hebron, Universitat Autònoma de Barcelona, Barcelona, Spain

**Keywords:** High mobility group box protein 1, Biomarkers, Multiple sclerosis

## Abstract

**Background:**

High mobility group box protein 1 (HMGB1) is a transcriptional regulator that is receiving increasing attention in autoimmune disorders including multiple sclerosis (MS). Here, we investigated the role of HMGB1 in the peripheral blood compartment from MS patients.

**Methods:**

*HMGB1* mRNA expression levels were determined by PCR in peripheral blood mononuclear cells (PBMC) of 29 healthy controls and 57 untreated MS patients (26 with relapsing-remitting MS - RRMS, 13 with secondary progressive MS - SPMS, and 18 with primary progressive MS - PPMS). HMGB1 protein levels were measured by ELISA in serum samples from 18 HC and 37 untreated MS patients (13 with RRMS, 14 with SPMS, and 10 with PPMS).

**Results:**

*HMGB1* expression levels were increased in PBMC from the whole MS group compared with controls (*P* = 0.03). Further stratification of the MS group revealed higher expression levels in PBMC from patients with relapse-onset MS, and differences were statistically significant for RRMS patients compared with PPMS patients and controls (*P* = 4 × 10^−5^ and *P* = 0.005, respectively) and also for SPMS patients compared with PPMS patients (*P* = 0.001). HMGB1 serum levels were increased in the whole MS group compared with controls (*P* = 2 × 10^−4^). In MS clinical forms, the highest HMGB1 serum levels were observed in RRMS patients, and differences were statistically significant compared to PPMS patients (*P* = 5 × 10^−5^), SPMS patients (*P* = 0.001), and controls (*P* = 0.001).

**Conclusions:**

These results point to a role of HMGB1 mRNA and protein levels as disease activity biomarkers to discriminate the more inflammatory relapse-onset MS forms, particularly RRMS, from the less inflammatory PPMS form of the disease.

**Electronic supplementary material:**

The online version of this article (doi:10.1186/s12974-015-0269-9) contains supplementary material, which is available to authorized users.

## Background

High mobility group box protein 1 (HMGB1) is a DNA-binding non-histone protein that acts as a transcriptional regulator and nucleosomal stabilizer [1,2]. It is released from activated immune cells, necrotic cells, and apoptotic cells and during secondary necrosis [3]. HMGB1 may drive pro-inflammatory responses through binding to cell-surface receptors such as the receptor of advanced glycation end products (RAGE) and the Toll-like receptors (TLR)-2, TLR-4, and TLR-9 [3]. Increasing evidence exists for a role of HMGB1 in autoimmune disorders. In this context, recent studies have shown associations between HMGB1 and rheumatoid arthritis [4], systemic lupus erythematosus [5], psoriasis [6], and Sjögren’s syndrome [7]. In multiple sclerosis (MS), HMGB1 expression was found to be upregulated in brain active lesions from patients [8]. In the animal model of MS, experimental autoimmune encephalomyelitis (EAE), neutralization of HMGB1 ameliorated clinical severity, reduced central nervous system pathology, and blocked pro-inflammatory cytokine production [9,10].

Based on these observations, in the present study, we aimed to investigate a potential role of HMGB1 in the peripheral blood compartment by determining its expression in peripheral blood mononuclear cells (PBMC) and circulating levels of HMGB1 in MS patients with different clinical forms and healthy controls.

## Methods

### Determination of *HMGB1* expression levels in PBMC by real-time PCR

mRNA expression levels for HMGB1 were determined in PBMC from 57 untreated MS patients and 29 healthy controls (HC). The MS group comprised 26 patients with relapsing-remitting MS (RRMS), 13 patients with secondary progressive MS (SPMS), and 18 patients with primary progressive MS (PPMS). Table 1 shows a summary of demographic and clinical characteristics of MS patients and controls included in the study.

**Table 1:** **Demographic and baseline clinical characteristics of MS patients and healthy controls included in the** ***HMGB1*** **expression study**

**Baseline characteristics**	**HC**	**MS** ^**d**^	**RRMS**	**SPMS**	**PPMS**
Number of patients	29	57	26	13	18
Age (years)	40.0 (9.1)	43.0 (9.9)	36.8 (7.8)	45.7 (6.5)	49.9 (9.5)
Female/male (% women)	13/16 (44.8)	31/26 (54.4)	16/10 (61.5)	8/5 (61.5)	7/11 (38.9)
Duration of disease (years)	-	9.2 (6.0)	5.7 (4.5)	10.9 (6.3)	10.6 (6.0)
EDSS^a^	-	3.9 (2.0-5.9)	1.9 (1.0-2.0)	4.2 (3.5-5.3)	5.3 (4.3-6.5)
Number of relapses^b^	-	1.4 (0.9)	1.7 (0.7)	1.1 (1.0)	-
Number of Gd-enhancing lesions^c^	-	0.9 (1.9)	1.0 (2.0)	0.7 (1.9)	-

Data are expressed as mean (standard deviation) unless otherwise stated. ^a^Data are expressed as mean (interquartile range). ^b^Refers to the number of relapses in the two previous years of blood extraction. ^c^Refers to the number of gadolinium-enhancing lesions at the time of blood extraction. ^d^One RRMS patient had a history of concomitant lupus anticoagulant. One SPMS patient had a past history of uveitis and breast cancer. One RRMS patient received corticosteroid treatment within the month prior to blood extraction for mRNA determination. Information is missing for eight patients (seven PPMS and one SPMS). MS: refers to the whole group of MS patients. EDSS: Expanded Disability Status Scale. RRMS: relapsing-remitting MS. SPMS: secondary progressive MS. PPMS: primary progressive MS. HC: healthy controls.

PBMC were isolated by Ficoll-Isopaque density gradient centrifugation (Gibco BRL, Life Technologies LTD, Paisley, UK) and stored in liquid nitrogen until used. Total RNA was extracted from PBMC using an RNeasy kit (Qiagen, Santa Clarita, USA) and cDNA synthesized using the High Capacity cDNA Archive Kit (Applied Biosystems, Foster City, CA, USA). *HMGB1* transcripts were determined with TaqMan® gene expression assays (Hs01590761_g1; Applied Biosystems). The housekeeping gene glyceraldehyde-3-phosphate dehydrogenase (*GAPDH*) was used as an endogenous control (Applied Biosystems). Assays were run on the ABI PRISM® 7900HT system (Applied Biosystems) and data were analyzed with the 2^−∆∆CT^ method [11]. Results were expressed as fold change in gene expression in MS patients or clinical forms of MS relative to healthy controls (calibrators).

### Quantification of HMGB1 levels in serum samples by ELISA

Serum levels of HMGB1 were measured in serum samples from 37 untreated MS patients and 18 healthy controls. The MS group included 13 patients with RRMS, 14 patients with SPMS, and 10 patients with PPMS. Table 2 summarizes demographic and clinical characteristics of MS patients and controls included in this part of the study. Eleven patients with RRMS (84.6%), five patients with SPMS (35.7%), and eight patients with PPMS (80%) were also used in the HMGB1 expression study. None of the 14 healthy controls included in the ELISA study was used in the expression study. Demographic and clinical characteristics were comparable for all variables between both cohorts (Additional file 1: Table S1).

**Table 2:** **Demographic and baseline clinical characteristics of MS patients and healthy controls included in the ELISA study**

**Baseline characteristics**	**HC**	**MS** ^**d**^	**RRMS**	**SPMS**	**PPMS**
Number of patients	18	37	13	14	10
Age (years)	42.8 (10.6)	43.6 (9.7)	36.0 (8.0)	44.7 (7.6)	51.9 (6.5)
Female/male (% women)	9/9 (50.0)	22/15 (59.5)	8/5 (61.5)	9/5 (64.3)	5/5 (50.0)
Duration of disease (years)	-	8.3 (5.0)	6.1 (4.4)	8.8 (4.0)	10.1 (5.6)
EDSS^a^	-	3.9 (2.3-5.3)	2.2 (1.5-3.0)	4.5 (4.0-5.1)	5.4 (4.3-6.5)
Number of relapses^b^	-	1.1 (0.9)	1.6 (0.8)	0.6 (0.8)	-
Number of Gd-enhancing lesions^c^	-	1.4 (2.8)	1.7 (3.2)	1.0 (2.2)	-

Data are expressed as mean (standard deviation) unless otherwise stated. ^a^Data are expressed as mean (interquartile range). ^b^Refers to the number of relapses in the two previous years of blood extraction. ^c^Refers to the number of gadolinium-enhancing lesions at the time of blood extraction. ^**d**^One SPMS patient had a history of concomitant gastritis. One RRMS patient received corticosteroid treatment within the month prior to blood extraction for protein determination. Information is missing for five patients (four PPMS and one SPMS). MS: refers to the whole group of MS patients. EDSS: Expanded Disability Status Scale. RRMS: relapsing-remitting MS. SPMS: secondary progressive MS. PPMS: primary progressive MS. HC: healthy controls.

Peripheral blood was collected by standard venipuncture and allowed to clot spontaneously for 30 min. Serum was isolated by centrifugation and stored frozen at −80°C until used. Levels of HMGB1 were measured in serum samples by means of a commercially available ELISA (Human HMGB1 ELISA; IBL International GMBH, Hamburg, Germany). All samples were measured in duplicate in undiluted serum samples. The intra-assay and inter-assay coefficients of variation were 2.1% and 1.1%, respectively.

### Statistical analysis

Statistical analysis was performed by using the SPSS 17.0 package (SPSS Inc, Chicago, IL) for MS Windows. Comparisons of mRNA expression levels and serum levels of HMGB1 between MS patients and healthy controls and between different clinical forms of MS, and correlations with clinical and radiological variables were performed by parametric and non-parametric tests depending on the applicability conditions. Bonferroni correction was used to correct the alpha level for multiple comparisons between MS patients with different clinical forms of the disease (alpha = 0.008).

### Ethics statement

The study was approved by the Hospital Universitari Vall d’Hebron ethics committee, and all patients gave their informed consent.

## Results

### *HMGB1* gene expression levels are increased in patients with relapse-onset forms of MS

**Figure 1:**
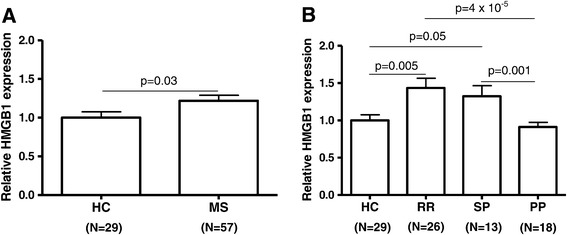
**Bar graphs comparing** ***HMGB1*** **mRNA expression levels in PBMCs from MS patients and healthy controls (A) and between different clinical forms of MS (B).** *HMGB1* expression was determined by real-time PCR using *GAPDH* as endogenous control. Results are expressed as fold change in *HMGB1* gene expression in MS patients relative to controls. Errors bars represent standard error of the mean. Number of individuals included in the study is shown in parentheses. HC: healthy controls. MS: whole group of multiple sclerosis patients. RR: relapsing-remitting MS. SP: secondary progressive MS. PP: primary progressive MS.

We first compared mRNA expression levels for *HMGB1* between the whole group of MS patients and healthy controls. As shown in Figure 1A, *HMGB1* expression was significantly increased in PBMC from MS patients compared to controls (*P* = 0.03). When we segregated patients on the basis of clinical forms (Figure 1B), *HMGB1* expression levels were higher in PBMC from patients with relapse-onset MS, and differences were statistically significant for RRMS patients when compared both with PPMS patients and controls (*P* = 4 × 10^−5^ and *P* = 0.005, respectively), and also for SPMS patients when compared with PPMS patients (*P* = 0.001). A trend towards increased mRNA expression levels of HMGB1 was observed in SPMS patients when compared with controls (*P* = 0.05). *HMGB1* expression levels were similar between the PPMS group and the control group (Figure 1B).

### HMGB1 serum levels are elevated in relapse-onset MS

**Figure 2:**
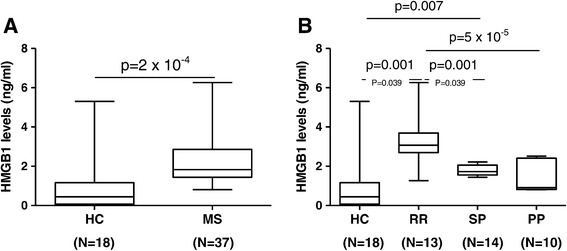
**Box plots showing serum levels of HMGB1 in the whole MS group and controls (A) and in MS patients with different clinical forms of MS (B).** Serum levels of HMGB1 were measured using a commercially available ELISA, as described in ‘Methods.’ For the sake of clarity, only significant *P* values are shown in the graphs. Number of individuals included in the study is shown in parentheses. Eleven patients with RRMS, five with SPMS, and eight with PPMS were also included in the *HMGB1* gene expression study. HC: healthy controls. MS: whole group of multiple sclerosis patients. RR: relapsing-remitting MS. SP: secondary progressive MS. PP: primary progressive MS.

As shown in Figure 2A, HMGB1 protein levels paralleled *HMGB1* mRNA expression levels, and serum levels were significantly increased in the whole MS group compared to the healthy control group (*P* = 2 × 10^−4^). When MS patients were stratified according to the different clinical forms, the highest HMGB1 serum levels were observed in patients with RRMS, and differences were statistically significant compared to PPMS patients (*P* = 5 × 10^−5^), SPMS patients (*P* = 0.001), and healthy controls (*P* = 0.001) (Figure 2B). In the SPMS group, statistically significant differences were only observed with the healthy control group (*P* = 0.007) but not with the PPMS group (*P* = 0.1). A trend towards increased serum levels of HMGB1 was observed in PPMS patients compared with controls (*P* = 0.01).

### Correlations between HMGB1 levels and clinical and radiological variables

No statistically significant correlations were observed between expression levels or protein levels of HMGB1 in patients with different clinical forms of MS and clinical variables (disease duration, number of relapses in the previous 2 years, and EDSS score at the time of blood collection) nor with radiological variables (number of gadolinium-enhancing lesions at the time of blood extraction) (data not shown).

## Discussion

HMGB1 has a dual role. In addition to contribute to nuclear homeostasis by acting as a transcriptional regulator and nucleosome stabilizer [1,2], HMGB1 can also play a role as a cytokine by being passively released from apoptotic/necrotic cells or actively secreted from monocytes, and subsequently binding to receptors such as RAGE, TLR-2, and TLR-4 [12,13]. In this context, HMGB1 has been shown to mediate pro-inflammatory cytokine production [14], T cell proliferation [15], and cell migration [16], actions that can certainly be pathogenically relevant for autoimmune disorders like MS.

In the present study, we found that MS patients showed increased mRNA and protein levels of HMGB1 as compared to healthy controls. Within the MS group, differences were driven by patients with relapse-onset MS, particularly by patients with RRMS. Both mRNA and protein levels for HMGB1 were clearly elevated in RRMS patients compared to controls and PPMS patients. At the protein level, differences were also observed between the RRMS and SPMS groups. It should be mentioned that one of the limitations of the study was the low number of patients in individual groups, although the statistical differences in mRNA and protein levels between groups were strong enough to survive Bonferroni correction.

Studies on HMGB1 are scarce in MS. HMGB1 expression was found to be upregulated in macrophages and microglia from actively demyelinating MS lesions [8]. In this same study, HMGB1 gene expression was also measured in cerebrospinal fluid (CSF) cells and PBMC from a small cohort of MS patients and non-inflammatory neurological controls, and mRNA expression levels were higher in CSF cells from MS patients compared with controls whereas no significant differences were observed in PBMC [8]. Factors mainly related to study design may well explain the discrepancies observed with the present study: inclusion of a subgroup of RRMS patients during relapse, lack of further stratification of the MS group into RRMS and SPMS patients for HMGB1 comparisons among groups, or lumping non-inflammatory neurological controls and healthy controls within a unique control group [8].

In SPMS patients, more prominent HMGB1 differences were observed at the mRNA than at the protein level. Whereas HMGB1 mRNA expression levels in SPMS patients were similar to those seen in RRMS patients and significantly higher compared to PPMS patients, HMGB1 protein levels were lower in the SPMS group than in the RRMS group and significant differences were lost with PPMS patients. Although the reasons for these differences are possibly manifold, the inclusion of a largely independent cohort of SPMS patients (64%) with less active disease for HMGB1 protein quantification may have contributed to the finding of lower protein levels compared to mRNA expression levels in this group of patients (mean number of relapses in the two previous years: 0.6 in the SPMS cohort used for protein determination versus 1.1 in the SPMS cohort used for mRNA quantification).

Finally, the group of patients with PPMS contributed little to the significant differences observed in mRNA and protein levels of HMGB1 between MS patients and control individuals. In fact, MS patients having this less inflammatory form of the disease behaved largely similar to the healthy control group in terms of HMGB1 mRNA and protein levels.

In conclusion, these results point to a role of HMGB1 in MS, particularly in patients with RRMS and SPMS. The mRNA expression levels in PBMC or serum levels of HMGB1 may be used as disease activity biomarkers to discriminate the more inflammatory relapse-onset forms of MS from the less inflammatory PPMS form of the disease.

## Additional file

Additional file 1: Table S1. Comparisons for demographic and clinical variables between the two cohorts of patients included in the study.
