# Supplementary material for: Role of high mobility group box protein 1 (HMGB1) in peripheral blood from patients with multiple sclerosis
Source: J Neuroinflammation. 2015 Mar 11;12:48. doi: 10.1186/s12974-015-0269-9 (PMC4359557; doi:10.1186/s12974-015-0269-9)
Supplement: Additional file 1: Table S1. — Comparisons for demographic and clinical variables between the two cohorts of patients included in the study. [file 12974_2015_269_MOESM1_ESM.doc]

**Additional file: 1 Table S1**. Comparisons for demographic and clinical variables between the two cohorts of patients included in the study.

| Baseline characteristics | HC | MS | RRMS | SPMS | PPMS |
| --- | --- | --- | --- | --- | --- |
| Age | p=0.212 | p=0.609 | p=0.903 | p=0.946 | p=0.621 |
| Gender | p=0.730 | p=0.628 | p=0.881 | p=0.699 | p=0.569 |
| Duration of disease | - | p=0.683 | p=0.771 | p=0.606 | p=0.980 |
| EDSS | - | p=0.913 | p=0.350 | p=0.402 | p=1.000 |
| Number of relapses | - | p=0.166 | p=0.720 | p=0.185 | - |
| Number of Gd-enhancing lesions | - | p=0.502 | p=0.786 | p=0.724 | - |

Data represent p-values resulting from the comparisons for the different demographic and clinical variables and for each clinical form between the two cohorts of patients using chi-square tests (gender) and Mann-Whitney tests (remaining variables). EDSS: Expanded Disability Status Scale. MS: refers to the whole group of MS patients. RRMS: relapsing-remitting MS. SPMS: secondary progressive MS. PPMS: primary progressive MS. HC: healthy controls.
